# AI Models for Surgical Decision Support in Spontaneous Intracerebral Hemorrhage: A Systematic Review in Relation to Trials and Guidelines

**DOI:** 10.1007/s12028-026-02501-7

**Published:** 2026-04-06

**Authors:** Helbert de Oliveira Manduca Palmiero, Carlos Gilberto Carlotti, Eberval Gadelha Figueiredo

**Affiliations:** Division of Neurosurgery, University of São Paulo Medical School, Dr. Enéas de Carvalho Aguiar Ave., 255, Room 5083, São Paulo, 05403-000 Brazil

**Keywords:** Spontaneous intracerebral hemorrhage, Artificial intelligence, Machine learning, Surgical trials, Evidence mapping

## Abstract

**Abstract:**

Artificial intelligence (AI) applications for spontaneous intracerebral hemorrhage (ICH) are rapidly expanding, particularly in perioperative imaging analysis and surgical decision support. Because most predictive AI models are developed using independent clinical datasets, they are not expected to explicitly reproduce randomized trial protocols or guideline decision rules. We therefore conducted a descriptive systematic review and evidence-mapping study to crosswalk AI model inputs, predicted targets, and outputs to major surgical trials and clinical guidelines (ENRICH, MIND, MISTIE III, SWITCH, STICH II, CLEAR, and AHA/ASA), identifying areas of overlap where AI operationalizes trial-relevant constructs and areas where AI-derived predictors may be hypothesis-generating for future trial design. Of 37 records identified (31 from database searches and 6 from hand searches), 21 studies met eligibility criteria. Publications increased after 2021, peaking in 2024 (*n* = 5) and remaining in 2025 (*n* = 3). Most cohorts were single-center (13/21), mainly from China, the USA, and Germany. Inputs were predominantly non-contrast computed tomography (CT); one study used magnetic resonance imaging (MRI) for trajectory planning. Deep learning was the most common analysis method (14/21), followed by classical machine learning (4/21) and radiomics-based methods (2/21). AI applications focused on perioperative imaging tasks, including eligibility assessment, postoperative quality assurance, trajectory planning, workflow optimization, and treatment-effect modeling. Three studies demonstrated direct alignment with surgical trial thresholds (e.g., MISTIE/CLEAR), ten were indirectly aligned, and eight had no clear linkage. AI models add value to imaging-based perioperative assessment in ICH and frequently target constructs central to trial- and guideline-based decision-making, even when trial criteria are not explicitly encoded. This crosswalk clarifies where AI outputs overlap with trial- and guideline-relevant constructs and where AI-derived decision boundaries diverge, reinforcing measurement targets that can be benchmarked against existing evidence and identifying candidates for hypothesis-generating evaluation in future studies.

**Graphical Abstract:**

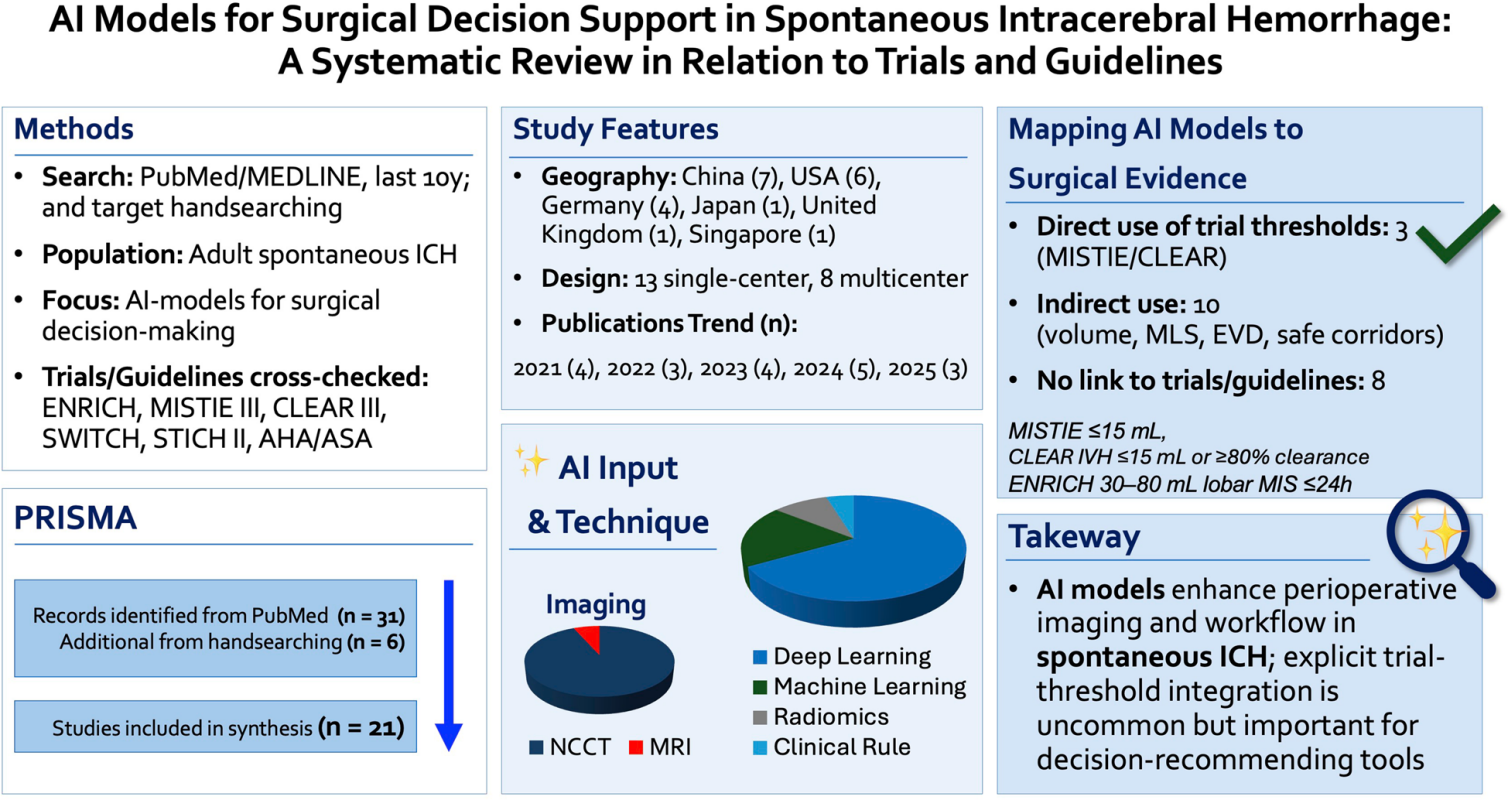

## Introduction

Intracerebral hemorrhage (ICH) is one of the most severe types of stroke, accounting for 10–15% of all cases and having early mortality rates of up to 40% [1]. Treatment decisions are guided by clinical criteria, including neurological status, age, and comorbidities, as well as findings from cranial computed tomography, such as hematoma size, location, intraventricular hemorrhage (IVH), hydrocephalus, and midline shift. Standard thresholds generally include supratentorial hematomas greater than 30 mL or infratentorial hematomas exceeding 15 mL [1–3].

Prognostic tools, such as the ICH score, offer further estimates of the 30-day mortality [4]. To determine the role of surgery, several randomized controlled trials (RCTs)—including ENRICH [2], MIND [5], MISTIE III [3], SWITCH [6], STICH II [7], and CLEAR [8]—have evaluated craniotomy, minimally invasive surgery (MIS), decompressive craniectomy, and external ventricular drainage (EVD) with thrombolysis. Despite these advances, no clear consensus has been reached regarding the specific surgical indications or techniques that improve long-term outcomes.

Alongside these clinical trials, advances in artificial intelligence (AI) have opened new opportunities for surgical decision support. Machine learning (ML), particularly deep learning (DL), can automatically analyze raw computed tomography (CT) data to identify hematomas, estimate their size, locate their position, and predict outcomes with greater accuracy than traditional volumetric methods. Several recent studies have investigated the role of AI in key decision-making processes in ICH management, including screening and volumetric assessment, as well as postoperative quality assurance and complication risk prediction.

Most AI models applied to spontaneous ICH are trained on independent datasets and are not designed to replicate randomized trial protocols or guideline algorithms. However, for AI tools intended to inform or influence surgical decision-making, it is valuable to examine which variables these models emphasize and how their predicted outcomes relate to eligibility criteria and endpoints used in major surgical trials. Such a comparison contextualizes model behavior within existing evidence frameworks without implying that AI systems are expected to replicate randomized trial protocols or guideline thresholds. Therefore, this review systematically maps AI model inputs, outputs, and decision-support functions to the trial- and guideline-based evidence framework for spontaneous ICH, providing a descriptive crosswalk between contemporary AI research and established surgical evidence.

## Methods

A descriptive systematic review and evidence-preference mapping study were performed to determine if artificial intelligence (AI) models for intracerebral hemorrhage (ICH) align with the criteria and findings of major surgical trials and guidelines. As a preliminary step, we summarized the key randomized and guideline sources that guide surgical decision-making in ICH (Table 1). For each source, we extracted the main evidence statements, clinical scenario, recommended approach, treatment window, and technical objectives, which served as the reference framework for classifying AI studies.

**Table 1:** Evidence on surgical management of spontaneous intracerebral hemorrhage (ICH): randomized clinical trials and guidelines, including ENRICH [2], MIND [5], MISTIE III [3, 9], SWITCH [6], STICH II [7], AHA/ASA guidelines [1], and CLEAR [8]

Evidence	Clinical scenario	Suggested approach	Time of intervention	Technical objectives
ENRICH [2]: improved utility-weighted mRS at 180 days, effect driven by lobar subgroup	Lobar supratentorial ICH, ~30–80 mL, stable enough for MIS, without substantial thalamic or intraventricular extension	Early MIS evacuation (trans-sulcal parafascicular/endoscopic) at experienced center	≤ 24 h from last-known-well; protocol goal ~8 h when feasible	Maximal safe evacuation; aim for small residual (~ < 15 mL when feasible)
ENRICH [2] subgroup: no benefit in anterior basal ganglia; MIND [7]: neutral primary endpoints	Supratentorial ICH (including deep/anterior basal ganglia hemorrhage), 20–80 mL, otherwise MIS-eligible	MIS evacuation in trial/selected cases; caution about benefit	≤ 24–72 h depending on protocol (ENRICH ≤ 24 h; MIND allowed ≤ 72 h)	Substantial clot reduction; shared decision-making regarding function
MISTIE III [3]: neutral primary; improved outcomes when residual < 15 mL; mortality trend favorable	Supratentorial ICH > 30 mL where catheter thrombolysis is feasible and team is experienced	Catheter-based aspiration + staged thrombolysis (MISTIE-style)	Catheter placement early, typically ≤ 72 h from ictus; alteplase 1.0 mg Q8h up to nine doses over ~1–3 days	End-of-treatment residual < 15 mL strongly associated with better outcomes
SWITCH [6]: primary endpoint narrowly missed; trend to fewer mRS 5–6; disability common	Severe deep ICH with malignant edema/ICP refractory to best medical therapy (coma, large MLS)	Decompressive craniectomy ± hematoma evacuation (salvage)	Urgent (hours) once ICP is refractory to maximal medical therapy; commonly within the first 1–2 days of clinical deterioration	Reduce mass effect/ICP; family counseling regarding disability
STICH II [7]: no overall functional benefit; possible small survival signal	Superficial lobar ICH without IVH, accessible to craniotomy, clinical deterioration or mass effect	Open craniotomy in selected/deteriorating cases; not routine for all	Surgery within 12 h after randomization; patients enrolled ≤ 48 h from ictus	Decompression/evacuation tailored to cortical site
MISTIE III post hoc [9]: MIS reduces MLS; MLS change fully mediates 30-day mortality reduction	Cross-cutting MIS quality target (any supratentorial MIS case)	Use evacuation strategy that limits midline shift (MLS) increase	Early MIS per trial windows (≤ 24 h in ENRICH; ≤ 72 h in MIND/MISTIE) to optimize MLS change	MLS < 3 mm at septum pellucidum and < 5 mm at pineal gland associated with lower 30-day mortality
AHA/ASA guideline [1]: class I recommendation to reduce mortality	Cerebellar hemorrhage (posterior fossa) with neurologic decline, brainstem compression, hydrocephalus, or > 3 cm	Immediate posterior fossa evacuation ± EVD	Immediate (hours) once criteria met; do not delay	Rapid decompression to prevent herniation/obstructive hydrocephalus
AHA/ASA guideline [1]: mortality reduction with EVD; functional benefit uncertain	IVH with obstructive hydrocephalus accompanying small–moderate ICH	EVD; consider intraventricular thrombolysis/endoscopic approaches at experienced centers	Immediate (hours) when hydrocephalus is present	CSF diversion; reduce shunt dependency (if neuroendoscopy)
CLEAR III [8]: no difference in primary endpoint (mRS ≤ 3 at 180 days); mortality reduced but with more survivors severely disabled. Alteplase safe (no ↑ rebleeding, ↓ ventriculitis). Trial clearance/stopping targets were opening of the 3rd and 4th ventricles, relief of IVH mass effect, and/or > 80% IVH removal	Small supratentorial ICH (≤ 30 mL) with obstrutive IVH involving the 3rd or 4th ventricles and a routinely placed EVD	Study design: EVD-based intraventricular irrigation: alteplase vs. saline	Immediate (EVD placement; thrombolysis within 72 h of onset)	EVD with intraventricular alteplase (rt-PA 1 mg Q8h, maximum 12 doses): open the 3rd/4th ventricles, relieve IVH mass effect, and/or achieve > 80% IVH removal

“Time of intervention” refers to the protocol-specified or guideline-recommended window for surgery or catheter placement (hours or days from ictus/last-known-well), with emergent interventions (posterior fossa, hydrocephalus, malignant ICP) prioritized independent of strict trial cutoffs

*ICH* intracerebral hemorrhage; *MIS* minimally invasive surgery; *IVH* intraventricular hemorrhage; *ICP* intracranial pressure; *MLS* midline shift; *EVD* external ventricular drain; *CSF* cerebrospinal fluid

A comprehensive PubMed/MEDLINE search was performed in September 2025. The a priori search string was:

“intracerebral hemorrhage”[tiab] AND (surg*[tiab] OR evacuation[tiab] OR endoscop*[tiab] OR “minimally invasive”[tiab]) AND (“artificial intelligence”[tiab] OR “machine learning”[tiab] OR radiomics[tiab] OR “deep learning”[tiab])

Searches were limited to the past decade to reflect the period of rapid growth in clinical AI publications. The reference lists of included studies were screened, and user-provided articles meeting the eligibility criteria were included. PubMed was chosen as the primary database owing to its comprehensive coverage of peer-reviewed biomedical and neurosurgical literature [10].

**Fig. 1:**
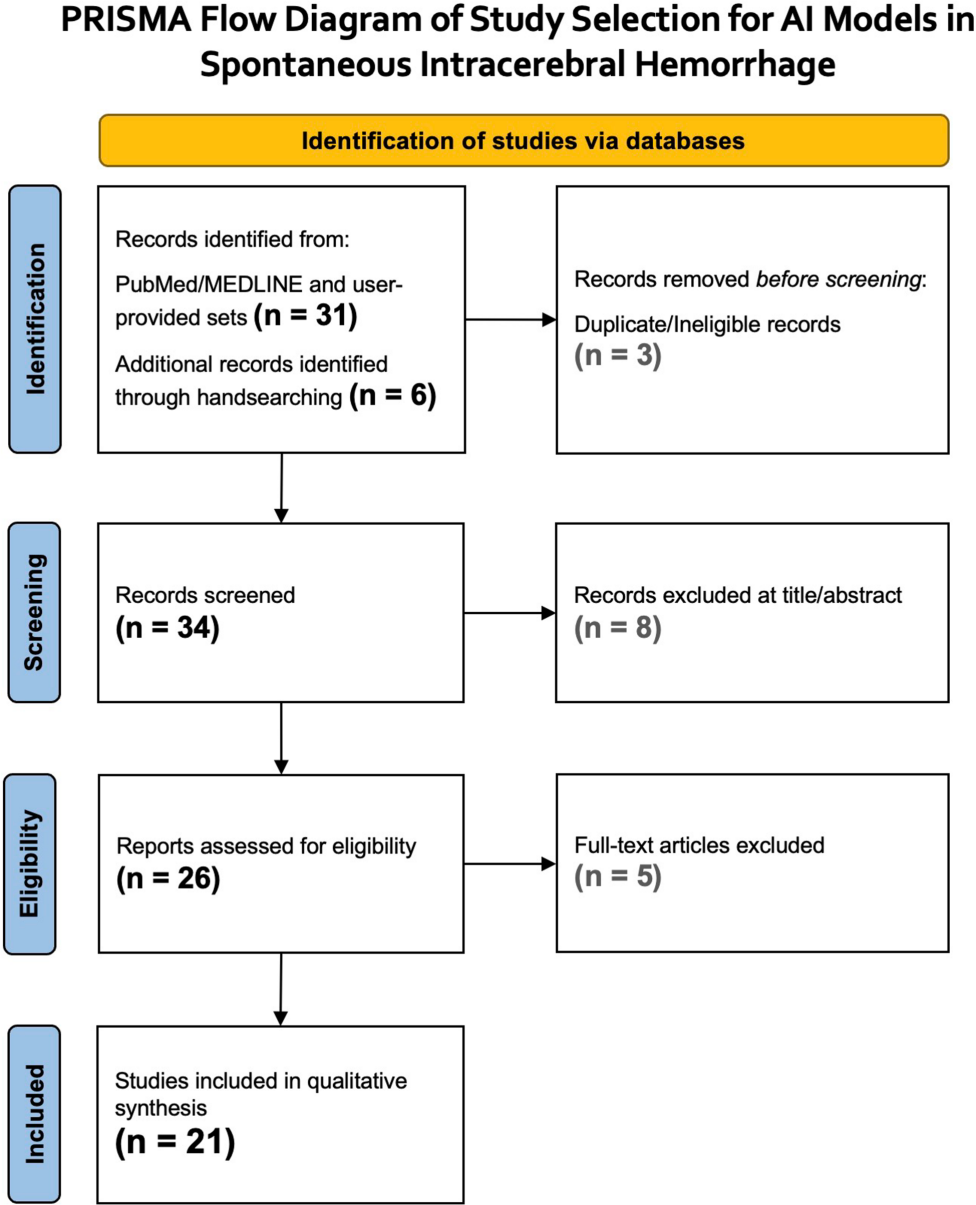
Flow diagram illustrating the identification, screening, eligibility assessment, and inclusion of studies evaluating artificial intelligence models for surgical decision-making in spontaneous intracerebral hemorrhage

To ensure transparency and reproducibility, the search was rerun prior to final submission, yielding 31 records. After removing duplicates and clearly ineligible studies, all retrieved records were screened at the title and abstract level. The updated numbers are reported in the revised Preferred Reporting Items for Systematic reviews and Meta-Analyses (PRISMA) flow diagram (Fig. 1). This update did not affect the final number of included studies.

In addition to the database search, targeted searching by hand was performed to ensure comprehensive coverage of influential and methodologically relevant AI studies in intracerebral hemorrhage surgery that might not be fully captured by keyword-based queries. This process involved screening the reference lists of all included articles, reviewing key publications from major author groups active in trial-related ICH imaging and AI research, and incorporating peer-reviewed articles supplied by users that met all prespecified eligibility criteria. Through this approach, six additional studies were identified and included in the qualitative synthesis and evidence mapping. These studies were evaluated using the same inclusion criteria, data extraction framework, and trial-alignment classification as the database-identified records.

### Inclusion Criteria (Prespecified)

Original, peer-reviewed human studies were eligible, whether retrospective, prospective, or derived from post-trial or registry analyses. Case series and case reports were considered when directly informing surgical decision-making or workflow. Populations had to include adult patients (≥ 18 years) with spontaneous intracerebral hemorrhage (ICH), with or without intraventricular extension. Studies required an artificial intelligence or machine learning component—such as radiomics-based approaches, classical machine learning algorithms, deep learning, or explainable/causal ML—and a clear connection to surgical care. Relevant areas included patient selection, trajectory planning, postoperative quality assurance (e.g., residual volume, drain position, or midline shift), perioperative complication risk, treatment effect estimation, and workflow and timing of surgery. Outcomes had to be relevant to surgical management, including functional status (mRS at 90–180 days), mortality, re-hemorrhage, technical success thresholds (≤ 15 mL residual or ≥ 70% clot removal), changes in midline shift, or time-to-OR metrics.

### Exclusion Criteria

Reviews, editorials, letters, conference abstracts without full peer-reviewed manuscripts, and purely technical papers lacking clinical data were excluded. Studies without an AI component or those using only conventional statistical methods unrelated to surgical decision-making were deemed ineligible. Additional exclusions included secondary ICH (such as those from tumors, arteriovenous malformations, or trauma), subarachnoid, epidural, or subdural hemorrhage, pediatric-only cohorts, and non-English full-text articles.

Two reviewers independently screened the titles and abstracts, followed by full-text evaluation against the eligibility criteria. Discrepancies were resolved through consensus. The study selection process is summarized in the Preferred Reporting Items for Systematic Reviews and Meta-analyses (PRISMA) flow diagram [11] (Fig. 1).

### Reporting Standards

Study identification and selection were reported in accordance with the PRISMA framework (Fig. 1). Given the imaging-centric nature of the included AI studies and the absence of a finalized PRISMA-AI extension, we additionally employed the Checklist for Artificial Intelligence in Medical Imaging (CLAIM) 2024 Update [12] to structure the extraction and reporting of AI-specific study characteristics (e.g., data source, reference standard terminology, internal versus external testing, and transparency regarding model/software/data availability). CLAIM was used as a reporting and organizational tool rather than as a scoring system.

### Data Extraction

For each study, information was collected on citation and year, country/center, cohort characteristics (number of surgical versus total; procedure type—craniotomy, MIS, endoscopy, stereotactic, decompressive craniectomy), inputs (clinical variables; imaging—noncontrast CT/magnetic resonance imaging (MRI), IVH, midline shift, postoperative scans; and radiomics), AI method (classified as classical ML, deep learning, radiomics-based ML, or explainable/causal ML), primary and secondary outcomes (e.g., mRS, mortality, complications at 90/180 days, technical endpoints such as residual volume or ≥ 70% removal), validation strategy (internal CV, external testing, calibration), and any explicit encoding of randomized trial criteria. Data were recorded in a structured extraction sheet and standardized to ensure consistency across studies. Extraction items were selected to reflect the review question (trial-alignment) and to include key AI reporting areas emphasized by CLAIM, such as data provenance, reference standard, partitioning/testing strategy, and transparency about code, model, and data availability when mentioned. The PRISMA and CLAIM checklists are provided in the Supplementary Appendix.

To address the research question, each AI paper was mapped to key trials and guidelines—ENRICH [2], MIND [5], MISTIE III [3, 9], SWITCH [6], STICH II [7], AHA/ASA guidelines [1], and CLEAR [8]. The alignment was categorized as:

- Direct: explicit use of trial thresholds/datasets (e.g., MISTIE residual ≤ 15 mL or ≥ 70% removal, ENRICH lobar 30–80 mL ≤ 24 h, CLEAR-related IVH clearance metrics, such as > 80% removal and/or opening of the 3rd and 4th ventricles).
- Indirect: models addressing constructs underpinning trial evidence (e.g., volumetry, midline shift, catheter/drain coverage, trajectory planning, workflow/time-to-OR) without explicitly encoding trial criteria.
- None: prognostic or complication-focused models without surgical evidence linkage.

These categories are descriptive and non-hierarchical; “direct” indicates explicit use of trial criteria/datasets, not superior model quality or clinical performance.

Given the heterogeneity of populations, interventions, AI methods, and outcomes, we conducted a qualitative synthesis instead of a meta-analysis. Although the synthesis was qualitative, the review was conducted using an a priori question, eligibility criteria, and structured data extraction; therefore, we report the study selection and results following PRISMA conventions (Fig. 1), while recognizing the descriptive evidence-mapping elements of the approach. Results are presented descriptively by decision-support function (postsurgical prognosis, postoperative quality assurance, complication risk, eligibility/quality assurance, trajectory planning, treatment-effect modeling, workflow/timing) and by trial-alignment category (direct/indirect/none).

### Risk of Bias and Study Quality

A formal meta-analytic risk-of-bias assessment was not feasible owing to methodological heterogeneity among the included studies. Instead, study quality was evaluated on the basis of design characteristics, including whether the methodology was prospective or retrospective, had multicenter participation, had presence of external testing on an independent dataset, had calibration, and whether trial criteria were explicitly defined. This approach aligns with current guidelines for descriptive systematic reviews and evidence mapping in biomedical research. Because no human subjects were directly involved, ethics approval was not required.

## Results

### Study Selection and Temporal Trends

**Fig. 2:**
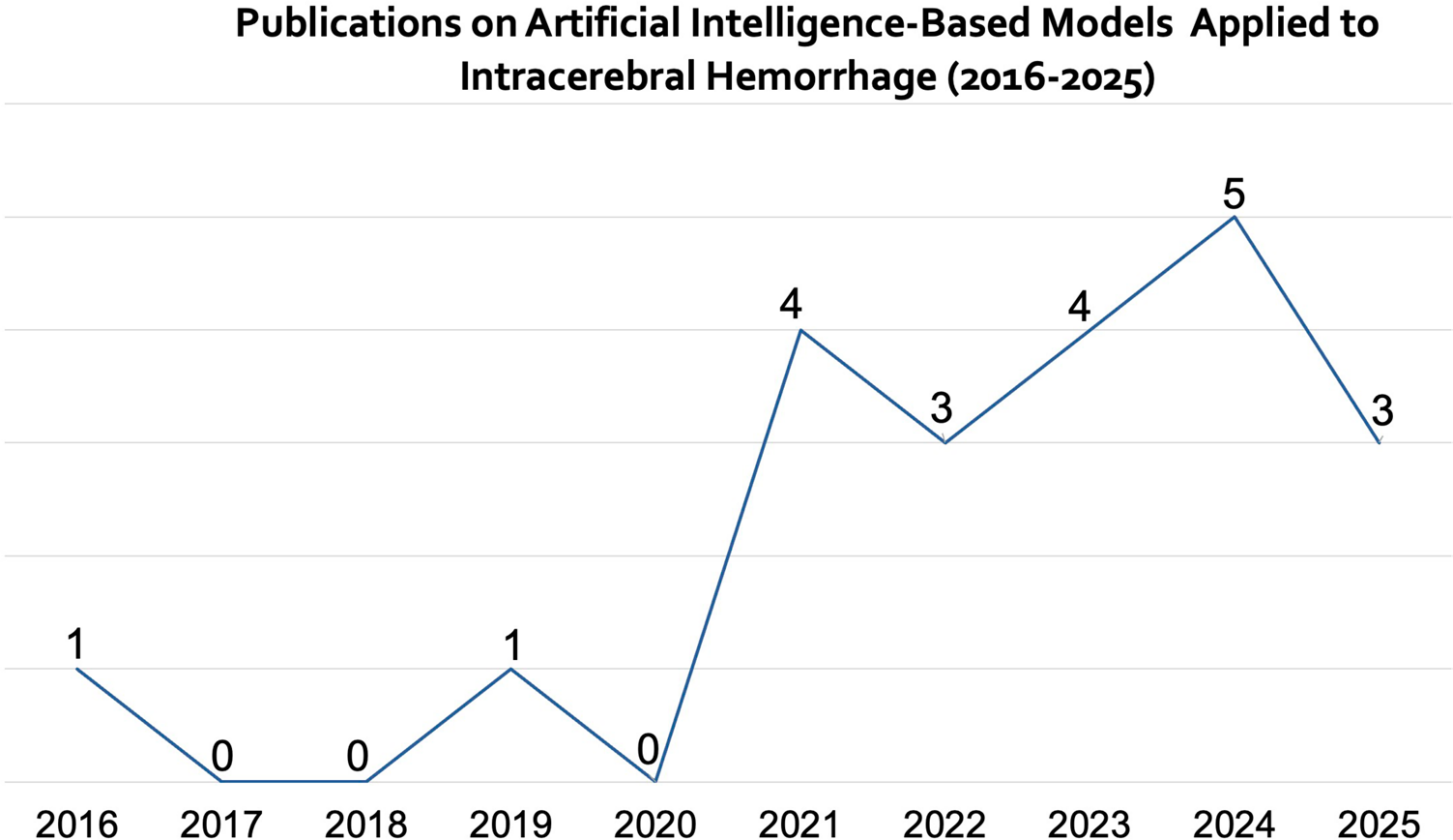
Annual number of publications on artificial-intelligence-based models applied to intracerebral hemorrhage from 2016 to 2025, showing a significant increase after 2020 with a peak in 2024

The PubMed search identified 31 records, and six additional studies were included through targeted searching. After full-text screening and removal of duplicates and ineligible records, 21 studies were included in the qualitative synthesis. Publications on AI models for ICH surgery increased significantly after 2021, reaching a peak in 2024 (*n* = 5) and remaining active from 2025 to date (*n* = 3) (Fig. 2).

### Study Characteristics

Most studies were single-center (13/21), with eight multicenter investigations. Geographically, China contributed seven studies, the USA six, Germany four, Japan one, the UK one, and Singapore one. Sample sizes ranged from a single case report to multicenter electronic medical record cohorts comprising more than 8000 patients. Imaging inputs were predominantly noncontrast CT; one study used MRI for surgical trajectory planning (Table 2).

**Table 2:** Summary of artificial-intelligence-based models for intracerebral hemorrhage surgery, including data inputs, analytical approaches, decision support functions, and alignment with evidence from randomized surgical trials and guidelines. Direct alignment denotes explicit use of trial criteria (e.g., MISTIE residual ≤ 15 mL, ≥ 70% removal; CLEAR-related IVH clearance metrics such as > 80% removal and/or opening of the 3rd and 4th ventricles), indirect alignment refers to models supporting workflow elements relevant to trial evidence (e.g., volumetry, MLS, trajectory), and “none” reflects prognostic or complication-focused models without surgical trial linkage

Study/year	Country/medical center	Data (*n* surgical/total; procedure: craniotomy, MIS, endoscopy, EVD)*	Inputs	AI method (machine learning, deep learning)	Decision support relevance	Trial alignment*
Scherer et al. [13]	Germany: Heidelberg University Hospital (single center)	*n* = 58 (study + validation); spontaneous supratentorial ICH (not surgery-only)	Imaging (CT)	Classical ML [Random forest (classical ML) segmentation]	Postoperative quality assurance—residual clot volume (≤ 15 mL target), drain position/coverage, Δmidline shift	Indirect
Ironside et al. [14]	USA: Columbia University Irving Medical Center (single center)	NA/397 (training *n* = 357; test *n* = 40), spontaneous ICH, post-evacuation CT excluded, EVD allowed	Imaging (CT)	Deep learning [convolutional neural networks (DL) segmentation]	Eligibility/quality assurance—automated hematoma volumetry	Indirect
Mansour et al. [15]	USA: University of Chicago (single center)	*n* = 19/286 eligible underwent MISTIE, 84% met ≤ 15 mL residual or ≥ 70% removal	Imaging (CT, segmentation features, volumetry)	Deep learning (3D deep neural network segmentation for ICH volume)	Postoperative quality assurance— residual clot volume (≤ 15 mL target), drain position/coverage, Δmidline shift	Direct (MISTIE III)
Katsuki et al. [16]	Japan: Suwa Red Cross Hospital (single center)	*n* = 140/140 (craniotomy or endoscopic), onset to surgery < 24 h	Clinical, imaging (CT)	Deep learning [deep learning framework (Prediction One)]	Postsurgical prognosis—predicts mRS/mortality after surgery (craniotomy/MIS) for risk counseling, benchmarking	None
Nawabi et al. [17]	Germany: Hamburg-Eppendorf, Charité Berlin, Münster (multicenter)	NA/520; acute spontaneous ICH; follow-up procedures recorded (craniectomy, intraventricular drainage placement)	Clinical, imaging (CT), radiomics	Radiomics-based ML (random forest ML using filter- and texture-derived image features)	Postsurgical prognosis—predicts mRS/mortality at discharge (not surgery-only)	None
Sharrock et al. [18]	USA: Johns Hopkins, UNC, University of Chicago (multicenter)	NA (MISTIE II *n* = 112; MISTIE III *n* = 500; pre-randomization diagnostic CT for trial workflow)	Imaging (CT, segmentation features, ICH + IVH + SDH)	Deep learning [2D and 3D deep neural network segmentation (DeepBleed)]	Eligibility/quality assurance—rapid ICH volume estimation for trial inclusion criteria and trial workflow	Direct (MISTIE II, MISTIE III)
Kok et al. [19]	UK: University of Nottingham; TICH-2 trial (multicenter)	NA (baseline noncontrast CT from TICH-2; *n* = 1732 annotated; test *n* = 174)	Imaging (CT, segmentation features, ICH + PHE + IVH)	Deep learning [3D nnU-Net variants; U-Net baseline (DL)]	Eligibility/quality assurance—automated segmentation/volumetry for clinical trials (ICH, PHE, IVH)	Indirect
Sharrock et al. [20]	USA: Johns Hopkins, UNC, University of Chicago (multicenter)	NA (diagnostic CT for trial eligibility/quality assurance)	Imaging (CT, segmentation features, ICH + IVH)	Deep learning [Bayesian deep learning (IV-Net/V-Net) with Monte Carlo dropout (DL)]	Eligibility quality assurance—automated ICH/IVH volumetry for trial eligibility and IVH burden/clearance assessment	Direct (MISTIE III, CLEAR)
Yu et al. [21]	China: Xuzhou Central Hospital (single-center)	NA (retrospective *n* = 512; prospective *n* = 50)	Imaging (CT)	Deep learning [dimension reduction UNet (DR-UNet) (DL)]	Eligibility/quality assurance—automated hematoma volumetry	Indirect
Cao et al. 2023 [22]	Germany/Italy: Charité Berlin, UMC Hamburg-Eppendorf, IRCCS Mondino Pavia (multicenter)	*n* = 0/1040; spontaneous ICH; excluded EVD or any surgical procedure	Imaging (CT, segmentation features, ICH + IVH)	Deep learning [external validation and retraining of DeepBleed (3D DL segmentation)]	Eligibility/quality assurance—automated ICH/IVH segmentation and volumetry for clinical trials	Indirect
Tong et al. 2023 [23]	China: First Affiliated Hospital of China Medical University (single center)	*n* = 351 (catheter puncture path planning; test *n* = 103)	Imaging (CT, segmentation features, IPH + IVH, volumetry, trajectory)	Deep learning [3D U-Net with multi-scale boundary aware module + consistency loss (DL)]	Trajectory planning—DL segmentation + safety corridors/trajectory selection for catheter/endoscopic access	Indirect
Lilieholm et al. [24]	USA: University of Wisconsin (single center)	*n* = 121 acquired; 27 T2-W suitable for MIS planning; MRI scans within 4 days of admission	Imaging (MRI T2-weighted, segmentation features, clot and edema, volumetry)	Deep learning [convolutional neural networks (separate CNNs for clot, edema)]	Trajectory planning—DL segmentation + safety corridors/trajectory selection for catheter/endoscopic access	Indirect
Zhang et al. 2023 [25]	China: Tongji Hospital, Shanghai East, Xinhua (multicenter)	*n* = 348 enrolled; *n* = 280 analyzed; all craniotomy evacuation	Clinical, imaging (CT, IVH, MLS, deep ICH), radiomics	Radiomics-based ML [radiomics + logistic regression (best with LASSO selection)]	Postsurgical prognosis— predicts mRS/mortality after surgery (craniotomy/MIS) for risk counseling, benchmarking	None
Elsheikh et al. [26]	Germany: University of Freiburg (single center)	*n* = 59 test scans/44 patients (drain present); post-MIS cohort	Imaging (CT, segmentation features, volumetry)	Deep learning [hierarchical patch-based CNN (DL)]	Postoperative quality assurance—residual clot volume (≤ 15 mL target), drain position/coverage, Δmidline shift	Indirect
Elsheikh et al. [27]	Germany: University of Freiburg (single center)	*n* = 68 MIS patients; independent test *n* = 44 (59 scans)	Imaging (CT, segmentation features, postoperative MIS)	Deep learning [CNN-based segmentation + ML classifiers for malposition (end-to-end pipeline)]	Postoperative quality assurance—residual clot volume (≤ 15 mL target), drain position/coverage, Δmidline shift	Indirect
Wang et al. [28]	China: Lu’an Hospital of Traditional Chinese Medicine (single center)	*n* = 609 hypertensive patients with ICH; all underwent craniotomy ≤ 72 h	Clinical, imaging (CT, baseline and follow-up re-hemorrhage), radiomics	Classical ML [SVM best (also RF, XGBoost, Logit tested) with SHAP interpretation]	Postoperative complication risk—early re-hemorrhage/complications using NCCT radiomics, clinical features	None
Li et al. [29]	China: Qingdao Municipal Hospital (single center)	*n* = 227 surgical patients (MIS aspiration + craniotomy)	Clinical, imaging (MLS, residual volume)	Classical ML [SVM, Decision Tree C5.0, ANN, logistic regression (SVM best)]	Workflow/timing—AI alerts and transfer optimization to reduce time-to-OR	None
Lim et al. [30]	Singapore: National University Hospital tertiary hospital (single center)	*n* = 282 total, 92 any surgery, 57 surgical evacuation, remainder medical/EVD	Clinical, imaging (CT)	Classical ML (SVM best; model calibration applied)	Treatment-effect modeling—PSM/SHAP/causal ML to estimate benefit of surgery vs. medical care	None
Gan et al. [31]	China: PLA General Hospital, Beijing (single center)	*n* = 347 CTs; supratentorial ICH surgical trajectory planning	Imaging (CT, segmentation features, trajectory)	Deep learning [deep learning (nnU-Net); automated reorientation + safety-zone corridors; optimization for path]	Trajectory planning—DL segmentation + safety corridors/trajectory selection for catheter/endoscopic access	Indirect
Xia et al. [32]	China: EMR database 15 centers, additional external 6 centers (multicenter)	*n* > 8000 cases, hypertensive ICH (EMR); outputs include surgical vs. nonsurgical plans	Clinical, imaging (CT, MLS, volumetry)	Deep learning [BERT-IDCNN-BiLSTM-CRF NER + KG-enhanced reasoning (explainable AI)]	Decision support(diagnosis + treatment plan including surgery)	None
Afreen et al. [33]	USA: Mount Sinai Health System (single center)	*n* = 1; supratentorial craniotomy with endoscopic evacuation + VP shunt	Imaging (CT)	Unspecified ML [regulatory-cleared AI detection (Viz.ai ICH Plus)]	Workflow/timing—AI alerts and transfer optimization to reduce time-to-OR	None

*“*n* surgical/total,” the first number is patients who had surgery, the second is all those who were included. Trial alignment: direct (explicit trial criteria), indirect (surgical workflow, not explicit trial criteria), and none (prognosis/complication focus). Trial names refer to ENRICH, MISTIE III, CLEAR, STICH II, SWITCH, and the AHA/ASA guidelines

*ANN *artificial neural network, *AUC* area under the ROC curve, *CNN* convolutional neural network, *CT* computed tomography, *DL* deep learning, *EMR* electronic medical record, *EVD* external ventricular drain, *ICH* intracerebral hemorrhage, *IVH* intraventricular hemorrhage, *MLS* midline shift, *ML* machine learning, *MIS* minimally invasive surgery, *MRI* magnetic resonance imaging, *RF* random forest, *SHAP* SHapley Additive exPlanations, *SVM* support vector machine

### AI Methods and Inputs

Deep learning was the most common analytic approach (14/21 studies), followed by classical machine learning (4/21), radiomics-based machine learning (2/21), and a regulatory-approved but otherwise unspecified AI tool in a single study (1/21). Inputs were primarily imaging-based and most often included automated segmentation or volumetry, assessment of intraventricular hemorrhage burden, and midline shift, with a minority of studies integrating limited clinical variables.

### Decision-Support Functions

The included models addressed a range of perioperative tasks. The most frequent applications involved eligibility assessment and postoperative quality assurance—such as measuring residual hematoma volume, drain position or coverage, and changes in midline shift. Other functions included planning trajectories for catheter or endoscopic access, predicting postoperative outcomes, optimizing workflow and timing (such as alerting or transfer prioritization), predicting complication risk, modeling treatment effects by comparing surgery with medical management, and offering comprehensive decision support or plan recommendations. Overall, imaging-based perioperative tasks were dominant, while fully integrated decision-support models remained uncommon.

### Alignment with Randomized Trials and Guidelines

Using predefined criteria, three studies demonstrated *direct* anchoring to surgical trial evidence by explicitly incorporating thresholds from MISTIE III and/or CLEAR III (e.g., residual volume ≤ 15 mL, ≥ 70% clot removal, or CLEAR-related IVH clearance targets such as > 80% removal and/or opening of the 3rd and 4th ventricles). Ten studies were *indirectly* anchored, addressing trial-relevant constructs such as volumetry, midline shift control, catheter or drain coverage, trajectory planning, or workflow and time-to-intervention metrics without explicitly encoding trial cutoffs. Eight studies found *no clear association* with surgical trial or guideline criteria, focusing instead on prognosis, complication risk, workflow optimization, or general decision support. Notably, no models explicitly implemented enrollment or timing thresholds from ENRICH, STICH II, or SWITCH in an end-to-end manner (Table 3).

**Table 3:** Alignment of artificial-intelligence-based models with major intracerebral hemorrhage surgical trials and guidelines

Trial/guideline	Surgical criteria	Direct alignment (AI model explicitly encodes or validates these criteria)
Minimally Invasive Surgery Plus rt-PA for ICH Evacuation (MISTIE III, phase III) [1, 2]	Supratentorial ICH, 30–80 mL, MIS + rt-PA; ≤ 72 h, targets: residual ≤ 15 mL or ≥ 70% removal	AI volumetry validating residual ≤ 15 mL or ≥ 70% removal using trial datasets
Minimally Invasive Surgery vs. Medical Management Alone for ICH (MIND, randomized clinical trial) [3]	Supratentorial ICH, 20–80 mL; MIS with Artemis; surgery within 72 h	AI replicating MIS clot reduction criteria directly
Early Minimally Invasive Removal of ICH (ENRICH, 2023) [4]	Lobar ICH, 30–80 mL, MIS endoport (BrainPath), ≤ 24 h from onset	AI explicitly encoding lobar location and trial volume/timing thresholds
Swiss Trial of Decompressive Surgery in Deep ICH (SWITCH, 2023) [5]	Deep ICH (basal ganglia, thalamus), 30–100 mL, GCS 8–13, surgery within 72 h, decompressive craniectomy plus best medical treatment	AI models applying deep location criteria or decompression endpoints
Surgical Trial in Lobar ICH (STICH II, 2013) [6]	Superficial lobar ICH, 10–100 mL, ≤ 48 h, no IVH	AI models explicitly applying lobar, volume, or IVH exclusion criteria
Clot Lysis: Evaluating Accelerated Resolution (CLEAR III, phase III) [7]	Small ICH (≤ 30 mL) with obstructive IVH involving the 3rd/4th ventricles, existing EVD; alteplase vs. saline, symptom onset within 24 h of diagnostic CT	AI volumetry supporting CLEAR-related IVH burden/clearance assessment on trial datasets (> 80% removal and/or opening of the 3rd and 4th ventricles)
AHA/ASA guidelines (2022 ICH Management Update) [8]	Guideline-based: early lobar surgery with mass effect; MIS reasonable; consider MLS, volume, and comorbidities	AI models explicitly applying guideline thresholds (rare)

*AI* artificial intelligence, *ICH* intracerebral hemorrhage, *IVH* intraventricular hemorrhage, *MIS* minimally invasive surgery, *MLS* midline shift

## Discussion

There is a growing number of publications evaluating AI-based models for analyzing spontaneous ICH in clinical practice. In our review, we identified 21 studies that met strict eligibility criteria, averaging  approximately four publications annually over the past 5 years (four in 2021, three in 2022, four in 2023, five in 2024, and 3 in 2025 so far). This growth parallels the broader adoption of large language model (LLM) tools, such as ChatGPT and Gemini, which were introduced in late 2022. The widespread accessibility of AI through prompt-based interfaces seems to have accelerated the increased use of these technologies for clinical data analysis, including in the domain of spontaneous ICH.

Geographically, the studies primarily originated from major AI players, including the USA and China, with additional contributions from Germany, Japan, and Singapore. The cohorts ranged from single-case reports to multicenter electronic medical record datasets involving over 8000 patients, showing both the diversity and scope of available data sources. Recognizing concerns about selection bias in AI training caused by nonuniform data inputs, our review emphasizes that the included studies utilized a variety of clinical and imaging datasets. This variation may reduce, to some degree, the risk of overfitting or biased results from narrowly focused patient groups.

Regarding methodology, most studies relied exclusively on imaging inputs, predominantly noncontrast CT, with only one study using MRI for trajectory planning [34]. A smaller group combined imaging features with limited clinical variables. Deep learning frameworks were the most common analytical approach (14/21 studies), while the remainder employed classical machine learning or radiomics-based methods. Since deep learning is a subset of machine learning, these findings indicate that machine learning remains the primary analysis method in this field. In addition, all studies used retrospective data, indicating that AI tools were not applied for real-time prediction but for analyzing existing clinical datasets. This is particularly important, considering the broader challenges of prospective predictive AI, where factors such as numerous variables, randomness, and principles from game theory can reduce predictive accuracy. In contrast, retrospective analyses of real-world clinical data, as in the reviewed studies, can provide more reliable insights.

The identified models addressed a variety of perioperative tasks, including postoperative quality assurance (such as residual hematoma volume, drain position, and changes in midline shift), trajectory planning for catheter or endoscopic access, postsurgical prognosis, workflow optimization and timing, complication-risk prediction, and treatment-effect modeling. The most common applications focused on imaging-derived surgical endpoints—specifically residual hematoma volume, drain positioning, and midline shift dynamics—highlighting the fundamental role of imaging in postoperative evaluation. This pattern aligns with the historical development of AI, where imaging-based outcome assessment has been the earliest and most consistent application.

### Alignment with Surgical Trials and Guidelines

Most AI models for spontaneous ICH are developed independently of randomized trial protocols and are not expected to explicitly reproduce trial eligibility criteria or guideline decision rules. Accordingly, the absence of direct trial encoding should not be interpreted as a deficiency. Instead, the purpose of this review was to provide a descriptive crosswalk between AI-derived inputs and outputs and the constructs used in major surgical trials and guidelines. Of the 21 included studies, 3 demonstrated direct evidence anchored to randomized surgical trials, 10 were indirectly anchored through trial-relevant constructs, and 8 had no comparison with trial or guideline criteria.

Across the included studies, AI models most frequently targeted variables aligned with trial- and guideline-relevant constructs, including hematoma and intraventricular hemorrhage volumetry, midline shift, residual clot volume after evacuation, catheter or drain placement, and workflow or time-to-intervention metrics. These variables correspond to technical endpoints and perioperative targets used in trials such as MISTIE III, CLEAR III, and ENRICH, even when trial-specific thresholds were not explicitly encoded. Fewer models addressed functional outcomes in a manner directly comparable to primary trial endpoints (e.g., mRS at 90–180 days), and explicit implementation of enrollment windows or decision thresholds from ENRICH, STICH II, or SWITCH was rare.

Several AI applications identified in this review automate measurements that are traditionally time-consuming or subject to interobserver variability, including volumetry, residual volume targets, IVH clearance, and midline shift. Automating these measurements may improve consistency in real-world practice, support quality assurance, and facilitate trial workflows or post hoc analyses. When AI systems are intended to recommend interventions (rather than automate measurements), it is helpful to specify a priori how outputs will be evaluated—for example, against trial/guideline constructs when appropriate and/or against independently learned decision boundaries—so that benefit, safety, and generalizability can be assessed transparently.

Beyond replicating established thresholds, AI models often incorporate additional imaging features, radiomic patterns, or multivariable risk profiles not explicitly used in existing trials. When consistently observed across independent cohorts, these predictors may inform future trial design by enabling eligibility enrichment, refining imaging-based surrogate endpoints, or identifying patient subgroups most likely to benefit from specific surgical strategies. In this context, the value of AI lies not in strict adherence to historical trial criteria but in providing data-driven insights that can be prospectively tested within rigorous trial frameworks.

### Limitations

This review has limitations that warrant acknowledgment. All included studies were retrospective, which restricted their ability to inform real-time clinical decision-making and left them vulnerable to the inherent biases of observational data. The heterogeneity of AI methodologies—ranging from classical machine learning to deep learning and radiomics-based approaches—precluded quantitative synthesis, necessitating a descriptive analysis rather than a meta-analysis. While the geographic diversity of studies suggests broad global interest, the predominance of single-center cohorts limits external generalizability. Only a few studies explicitly evaluated model outputs against trial- or guideline-defined constructs (e.g., enrollment criteria, technical targets, or prespecified endpoints), which constrained this crosswalk’s ability to compare AI-derived decision boundaries with established evidence frameworks (without implying that such concordance is required for AI model validity). Future prospective multicenter studies should prioritize external validation, calibration, and prespecified benchmarking against clinically meaningful outcomes (including trial endpoints where relevant), rather than assuming concordance with historical thresholds.

### Clinical Implications

#### Clinical Correlation with the Review findings

This review indicates that contemporary AI in ICH surgery is most effective when it focuses on imaging-derived perioperative endpoints—such as hematoma/IVH volumetry, drain detection/coverage, residual volume, and midline shift—because these variables are routinely obtained, time-sensitive, and tightly coupled to operative assessment. In this context, even models that are only indirectly aligned can offer valuable clinical support by improving measurement consistency, reducing interobserver variability, and standardizing postoperative quality assurance without dictating treatment decisions.

#### How Alignment Influences Real-World Decision-Making

The relevance of trial alignment depends on the intended clinical role. For tools used as bounded assistants (such as segmentation, volumetry, quality assurance, and workflow triage), the absence of explicit RCT thresholds does not diminish their value; clinicians can incorporate outputs into existing decision frameworks. For AI systems providing treatment recommendations (such as surgery/no surgery, timing, or technique), it is useful to specify how the decision boundary will be evaluated relative to existing evidence (including trials/guidelines when relevant) and/or independent performance targets. In addition, deployment should incorporate safeguards to prevent automation bias (e.g., human-in-the-loop review, transparent reporting of uncertainty, and ongoing monitoring). Importantly, strict adherence to historical trial criteria should not be seen as a ceiling on innovation; instead, it should serve as a reference baseline against which new strategies can be compared prospectively.

#### Need for Multimodal and Temporal Integration

A common gap across studies is the limited integration of nonimaging information. Surgical ICH decisions are inherently multimodal and time-dependent, involving neurological examinations, severity scales, physiology, deterioration trajectories, and time-to-intervention. Future systems should therefore go beyond imaging-only pipelines and intentionally integrate clinical, physiological, and temporal data with radiologic features and trial-defined targets, enabling models that mirror real-world bedside decision-making pathways rather than isolated imaging tasks.

#### Ethics, Translational Informatics, and Evidence-Based Implementation

Translating AI tools into neurocritical care requires more than just technical performance: models must undergo external testing on independent datasets, be monitored for performance drift, and be deployed with clear accountability and clinician oversight. Ethical considerations include interpretability proportional to risk, mitigation of automation bias, and transparency regarding model limitations and data provenance. Trial-grade repositories and benchmarking resources (including VISTA-ICH) can facilitate reproducible evaluation and external testing, helping shift AI from promising retrospective tools toward evidence-based instruments that can be safely integrated into surgical decision support.

#### Can AI Assist in Clinical Decision-Making for ICH Management, and Might it Eventually Reshape Surgical Indications in a Similar Way to CT Scanning?

Our synthesis suggests that while AI has shown promise in technical endpoints (such as volumetric precision and postoperative quality assurance), its integration into surgical decision frameworks remains incomplete. To advance, AI systems intended for higher-stakes decision support should move beyond isolated imaging outputs toward multimodal, time-aware modeling and be evaluated prospectively for clinical impact. For such systems, trial/guideline constructs can serve as useful external benchmarks, but models may also define independent decision boundaries that require separate validation for safety, benefit, and generability.

## Conclusions

In summary, contemporary AI research in spontaneous intracerebral hemorrhage is rapidly growing and making meaningful contributions to perioperative assessment, particularly in imaging-based tasks such as volumetry, drain assessment, residual clot evaluation, and midline shift analysis. Most models are appropriately developed from independent datasets and do not explicitly encode randomized trial protocols; nevertheless, many target constructs central to trial- and guideline-based surgical decision-making. This review maps AI inputs and outputs onto recognized evidence frameworks, clarifies where AI applies trial- and guideline-related constructs, and where it adds new predictors or decision points. For future systems offering treatment advice, ensuring safety will require prospective testing, external validation, calibration, and transparent governance. While trial- and guideline-level constructs can serve as benchmarks for comparison, agreement with them should not be automatically assumed or mandated.
